# Efficacy and safety of traditional Chinese medicine combined with routine western medicine for the asymptomatic novel coronavirus disease (COVID–19): A Bayesian network meta-analysis protocol

**DOI:** 10.1097/MD.0000000000021927

**Published:** 2020-08-28

**Authors:** Jiahao Wang, Xue Zhu, Yuying Sun, Xingcai Zhang, Wei Zhang

**Affiliations:** a First College of Clinical Medicine, Shandong University of Traditional Chinese Medicine; b Affiliated Hospital of Shandong University of Traditional Chinese Medicine, Jinan; c Agricultural and Rural Bureau of Hedong District, Linyi City, Shandong Province, PR China.

**Keywords:** asymptomatic infection, COVID–19, network meta-analysis, traditional Chinese medicine

## Abstract

**Background:**

The number of patients infected with novel coronavirus disease (COVID–19) has exceeded 10 million in 2020, and a large proportion of them are asymptomatic. At present, there is still no effective treatment for this disease. Traditional Chinese medicine (TCM) shows a good therapeutic effect on COVID–19, especially for asymptomatic patients. According to the search results, we found that although there are many studies on COVID–19, there are no studies targeting asymptomatic infections. Therefore, we design a network meta-analysis (NMA) to evaluate the therapeutic effect of TCM on asymptomatic COVID–19.

**Methods:**

We will search Chinese and English databases to collect all randomized controlled trials (RCTs) of TCM combined with conventional western medicine or using only TCM to treat asymptomatic COVID–19 from December 2019 to July 2020. Then, two investigators will independently filter the articles, extract data, and evaluate the risk of bias. We will conduct a Bayesian NMA to evaluate the effects of different therapies. All data will be processed by Stata 16.0 and WinBUGS.

**Results:**

This study will evaluate the effectiveness of various treatments for asymptomatic COVID–19. The outcome indicators include the time when the nucleic acid turned negative, the proportion of patients with disease progression, changes in laboratory indicators, and the side effects of drugs.

**Conclusion:**

This analysis will further improve the treatment of asymptomatic COVID–19.

**INPLASY registration number:**

INPLASY202070022.

## 1 Introduction

In 2020, COVID–19 broke out all over the world. At present, the number of infected patients has exceeded 10 million, and the number of deaths has exceeded 500,000. This epidemic severely damaged the world economy and people's health.^[1–3]^ Therefore, controlling the epidemic has become the most important task at present. The main treatments for this disease are antiviral, symptomatic treatment, and nutritional support treatment.^[4]^ However, we still have no specific drugs against the COVID–19, so the clinical efficacy is not satisfactory.^[5,6]^ Worldwide, patients with COVID–19 still increase by tens of thousands every day. We are facing the dilemma of a shortage of medical resources and imperfect treatment. Therefore, we urgently need to find safer and more effective treatment measures to improve the COVID–19 treatment plan for people in the epidemic center to control the spread of the epidemic more quickly.^[7]^

The COVID–19 patients were categorized into light, ordinary, and critical types based on the Corona Virus Disease 2019 Diagnosis and Treatment Program (Chinese Seventh Edition). And a large part of the population is asymptomatic infection. If the nucleic acid test was positive, and patients had no obvious symptoms, they were diagnosed as asymptomatic infectors. Studies have reported that the proportion of asymptomatic patients accounts for 30.8%.^[8]^ A statistical analysis of 174 children's infections in Wuhan Children's Hospital showed that asymptomatic or subclinical COVID-19 patients accounted for 30.5%, and all cases were family clustered.^[9]^

It is undeniable that asymptomatic patients are infectious, but their infectious strength and mode of infection need to be further scientifically studied.^[10–12]^ Studies with small sample sizes have shown that the viral load in the respiratory tract samples of asymptomatic patients is not much different from the diagnosed patients.^[13,14]^ Another survey data on contact transmission showed that the infection rate of COVID-19 confirmed patients was 6.3%, and that of asymptomatic cases was 4.11%.^[15]^ Epidemiological investigations revealed a cluster of outbreaks caused by asymptomatic infections, which made asymptomatic patients a threat to the spread of the epidemic.^[16,17]^

Research data shows that asymptomatic patients will have disease progression and even death.^[18]^ Our research team conducted a small sample survey about asymptomatic infections in Shandong Province, China. The results showed that among 33 asymptomatic COVID–19 patients, seven (21.21%) converted to light type and six (18.18%) to ordinary type. (Table 1 lists the prognostic information.)

**Table 1:**
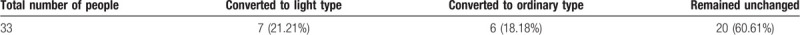
Prognosis information of patients.

**Figure 1:**
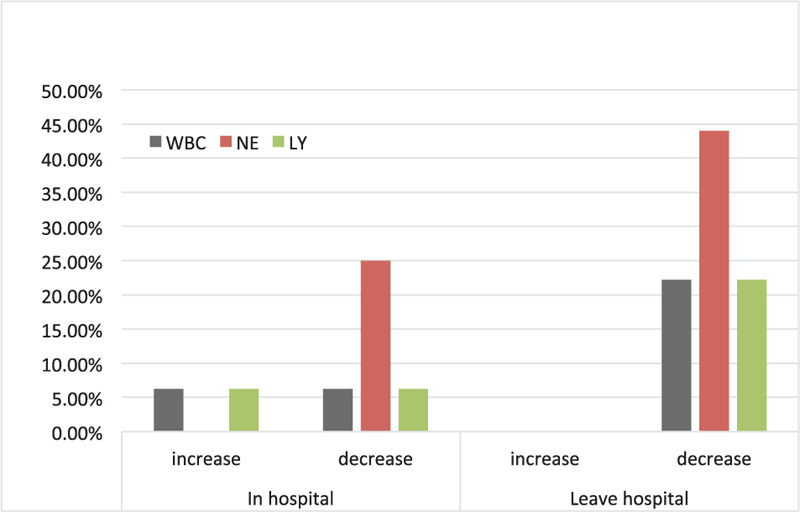
Proportion of patients with abnormal blood cell count. In hospital: At the time of admission; Leave hospital: At the time of discharge.

Our data also found that some patients had abnormal laboratory indicators, indicating that even if there were no symptoms, the patients still had immunosuppression (Table 2). Blood routine information at admission and discharge in Figure 1. The proportion of patients with abnormal blood cell count. Therefore, the prevention and treatment of asymptomatic infections have an impact on the control of the epidemic.^[19,20]^

**Table 2:**
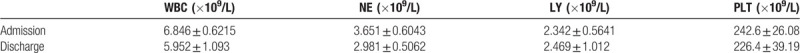
Blood routine information at admission and discharge.

China was the first country to discover the COVID–19, but now the epidemic in China has been controlled. This is the advantage of applying TCM. TCM neither aimed at specific symptoms of patients nor directly killed certain viruses. Its treatment principle is to improve the body's ability to fight viruses through the application of Chinese herbal medicine, thereby indirectly defeating the virus.^[21]^ This ability aims at all pathogenic microorganisms, even if this is a new virus, so it has a unique advantage for COVID–19, especially asymptomatic patients.^[22,23]^ In the epidemic, doctors in Wuhan applied Chinese herbal medicines such as Qingfei Paidu Decoction as a supplement to Western medicine, confirming that TCM can help accelerate the recovery of patients.^[24]^ Many Chinese patent medicines, such as Huoxiang Zhengqi, Lianhua Qingwen, Xuebijing, have been used in clinical practice and achieved a good curative effect.^[25]^ Colunga Biancatelli RML found that quercetin, a traditional Chinese medicine extract, has antiviral effects and can be used as preventive medicine for high-risk groups of COVID–19.^[26]^ The application rate of TCM was over 90%, and the effective rate was over 95%. Therefore, the effectiveness of TCM in the treatment of asymptomatic COVID–19 should be further studied.

## 2 Methods

We will use Bayesian NMA. Then we compliant PRISMA-P guidelines to conduct this study.

### 2.1 Study registration

This NMA has been registered on the International Platform of Registered Systematic Review and Meta-analysis Protocols (INPLASY), and the registration number is INPLASY202070022 (URL = https://inplasy.com/inplasy-2020-7-0022/).

### 2.2 Inclusion criteria

#### 2.2.1 Type of study

Those relevant RCTs about TCM for asymptomatic COVID-19 published in Chinese or English will be included.

#### 2.2.2 Participants

Patients diagnosed with asymptomatic COVID–19 infection. Patients with asymptomatic COVID–19 are those who have no relevant clinical symptoms but have a positive pathogenic test of respiratory tract specimens for COVID–19. Age, gender, race, nationality are not considered.

#### 2.2.3 Interventions

The treatment group used Chinese medicine as a supplement to western medicine treatment or just applied TCM alone. TCM includes lianhua qingwen, Huoxiang zhengqi, qingfei paidu, and other herb prescriptions. The control group used western medicine, such as antiviral therapy. There are no limits to dosage, usage, and course of treatment.

#### 2.2.4 Outcomes

Because the patients included in the study have no obvious symptoms, the main outcomes are safety and prognostic indicators, including the time when the nucleic acid turned negative, the proportion of patients with disease progression, changes in laboratory indicators, and side effects of drugs. It also includes the patient's mental state, psychology health, and other indicators.

### 2.3 Database and search strategy

We will comprehensively search the following electronic databases: Cochrane Library, PubMed, Web of Science, EMBASE, Chinese Biomedical Literature Database (SinoMed), Chinese National Knowledge Infrastructure (CNKI), Wanfang database, and VIP database from December 2019 to July 2020. The search strategy will be constructed in the form of Medical Subject Headings (MeSH) combine with keywords, including COVID–19, Asymptomatic Infection, traditional Chinese Medicine, Chinese Herbal Medicine, lianhua qingwen, Huoxiang Zhengqi, qingfei paidu, Anti-Virus, Random Control Trials, etc. (The retrieval scheme of the PubMed database is listed in Table 3.)

**Table 3:**
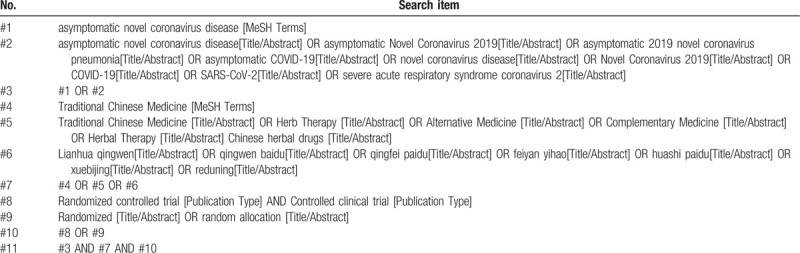
Detailed search strategy for PubMed.

### 2.4 Study selection and data extraction

We will retrieve all relevant papers from the database according to the above strategy, and then import the articles into endnote. Then two workers screened and extracted data separately. If there is a disagreement, resolve it through a third party. The information that needs to be extracted includes author, title, country, publication date, journal, random method, registration number, age, race, diagnostic criteria and sample size, intervention measures, and outcome indicators. If the necessary information is incomplete, we will consider contacting the original author.

### 2.5 Risk of bias assessment

The quality will be evaluated by two workers separately according to the Cochrane Collaboration Risk of Bias Tool.^[27]^ Seven items will be assessed, and the evaluation result is divided into “low,” “unclear,” and “high.”

### 2.6 Statistical analysis

We will use Stata 16.0 software and Markov chain-Monte Carlo (MC-MC) method for Bayesian mesh meta-analysis. Three Markov chains will be used for simulation, and the number of iterations is set to 50,000 times (the first 20,000 times are used to eliminate the influence of the initial value, and the last 30,000 times are used for sampling). Then, a network diagram will be drawn through Stata 16.0 software to show the direct and indirect comparison between different interventions. By calculating the relative odds ratio (RoR) and its 95% confidence interval (CI) to evaluate the consistency of each closed loop. If the lower limit of 95% CI is equal to 1, the consistency is good. If RoR is close to 1, it indicates that the direct evidence and the indirect evidence are consistent, and the fixed effect model will be used for analysis. Otherwise, it is considered that there is obvious inconsistency in the closed-loop, and the random effect model will be used for analysis.

Binary data will be expressed by odds ratio (OR) and 95% CI. *P* < .05 indicates that the difference is statistically significant. Then, the WinBUGS 1.4.3 software will be used to sort the efficacy of different interventions and record the area under the curve (the area under the curve is expressed as a percentage, the greater the percentage, the better the treatment effect). Finally, Stata 16.0 software will be used to draw an inverted funnel chart to evaluate whether the intervention measures have a small sample effect or publication bias, and a sensitivity analysis will be conducted.

### 2.7 Assessment of heterogeneity

The chi-square test will be used to assess heterogeneity. If the network *I*^2^ ≤ 50%, the heterogeneity shows small, and the fixed effect model will be applied. If the overall network *I*^2^ > 50%, the heterogeneity is obvious, then, we need to analyze the reasons for the heterogeneity. When heterogeneous factors are excluded, we then choose a random-effects model.

### 2.8 Subgroup analysis and sensitivity analysis

If the evidence is sufficient, subgroup analysis will be considered to seek the source of heterogeneity. Then, the sensitivity analysis will be performed by excluding every article. If the heterogeneity changes, the excluded article may be the reason for the heterogeneity.

### 2.9 Evaluation of publication bias and evidence quality

If the article has been researched more than 10 times, we will construct a comparison and correction funnel chart of the result indicators. On the premise that the funnel chart is symmetrical, the publication bias is not obvious; if the funnel chart is asymmetric, there may be publication bias. GRADE will be used as a reference to evaluate the quality of evidence, including the following five aspects: risk of bias, indirectness, inconsistency, imprecision, and publication bias.

## 3 Discussion

Since the discovery of COVID–19 in late 2019, it has spread globally at a terrifying speed and has caused a heavy blow to all countries. Up to now, the disease still lacks effective treatment methods, antiviral and other therapies are not effective, and vaccine development is also slow. With the advent of autumn and winter, the temperature becomes cold, and the epidemic is likely to develop to another peak. The COVID–19 patients were categorized into light, ordinary, and critical types. In addition, we define the type without clinical symptoms as asymptomatic infection. It has been proven that asymptomatic infection is contagious and has the possibility of disease progression. Therefore, the control of asymptomatic infection is an important part of the prevention of COVID–19, but there is still no publicly approved version of the treatment plan for asymptomatic infection. Some clinicians believe that antiviral treatment should be used, while others believe that asymptomatic infections do not require medical treatment. This is because the existing antiviral drugs have no obvious effect on the novel coronavirus, and we lack other effective treatment measures. Therefore, the treatment of asymptomatic infections is currently a global problem.

TCM has achieved satisfactory and unexpected therapeutic effects in China's fight against COVID–19. Since the first discovery of COVID–19 in Wuhan at the end of 2019, China has effectively controlled the epidemic with a series of measures and the help of TCM. In China, more than 95% of patients will choose TCM as a supplement while applying for conventional western medicine. Thousands of years ago, TCM has been applied to antiviral treatment. According to the theory of TCM, its therapeutic effect is not to have the ability to directly kill the virus but to improve the body's own ability to fight the virus, thereby indirectly killing the virus.

Network meta-analysis can compare the advantages and disadvantages of various treatments. According to our current search results, although researchers have done many investigations and studies on COVID–19, we have not yet found a Bayesian NMA on the evaluation of TCM combined with Western medicine for asymptomatic COVID–19. Therefore, we designed this study to rank different treatment methods and assess the efficacy and safety of TCM for asymptomatic COVID–19, to provide clinicians with a perfect treatment plan for the treatment of this disease.

Although network meta-analysis has many advantages, our research may still be unable to avoid some limitations and deficiencies, such as some publication biases that are difficult to rule out. We hope that more high-quality RCTs will be included to continuously improve the quality of evidence-based medicine and provide a better reference for the formulation of clinical treatment plans for asymptomatic COVID–19.

## Author contributions

**Conceptualization:** Jiahao Wang.

**Data curation:** Jiahao Wang, Xue Zhu, Xingcai Zhang.

**Formal analysis:** Yuying Sun, Xingcai Zhang.

**Methodology:** Jiahao Wang, Xue Zhu.

**Project administration:** Yuying Sun.

**Software:** Jiahao Wang, Yuying Sun.

**Writing – original draft:** Jiahao Wang, Yuying Sun.

**Writing – review & editing:** Jiahao Wang.
